# Discovery of Potential piRNAs from Next Generation Sequences of the Sexually Mature Porcine Testes

**DOI:** 10.1371/journal.pone.0034770

**Published:** 2012-04-06

**Authors:** Gang Liu, Bin Lei, Yan Li, Keya Tong, Yi Ding, Lifan Luo, Xuanyan Xia, Siwen Jiang, Changyan Deng, Yuanzhu Xiong, Fenge Li

**Affiliations:** 1 Key Laboratory of Pig Genetics and Breeding, Ministry of Agriculture and Key Laboratory of Agricultural Animal Genetics, Breeding and Reproduction of Ministry of Education, Huazhong Agricultural University, Wuhan, People’s Republic of China; 2 College of Science, Huazhong Agricultural University, Wuhan, People’s Republic of China; Kaohsiung Chang Gung Memorial Hospital, Taiwan

## Abstract

Piwi- interacting RNAs (piRNAs), a new class of small RNAs discovered from mammalian testes, are involved in transcriptional silencing of retrotransposons and other genetic elements in germ line cells. In order to identify a full transcriptome set of piRNAs expressed in the sexually mature porcine testes, small RNA fractions were extracted and were subjected to a Solexa deep sequencing. We cloned 6,913,561 clean reads of *Sus Scrofa* small RNAs (18–30 nt) and performed functional characterization. *Sus Scrofa* small RNAs showed a bimodal length distribution with two peaks at 21 nt and 29 nt. Then from 938,328 deep-sequenced small RNAs (26–30 nt), 375,195 piRNAs were identified by a *k*-mer scheme and 326 piRNAs were identified by homology searches. All piRNAs predicted by the *k*-mer scheme were then mapped to swine genome by Short Oligonucleotide Analysis Package (SOAP), and 81.61% of all uniquely mapping piRNAs (197,673) were located to 1124 defined genomic regions (5.85 Mb). Within these regions, 536 and 501 piRNA clusters generally distributed across only minus or plus genomic strand, 48 piRNA clusters distributed on two strands but in a divergent manner, and 39 piRNA clusters distributed on two strands in an overlapping manner. Furthermore, expression pattern of 7 piRNAs identified by homology searches showed 5 piRNAs displayed a ubiquitous expression pattern, although 2 piRNAs were specifically expressed in the testes. Overall, our results provide new information of porcine piRNAs and their specific expression pattern in porcine testes suggests that piRNAs have a role in regulating spermatogenesis.

## Introduction

Spermatogenesis is a complex process of cellular divisions and developmental changes that occur within the seminiferous tubules of the testes [1]. Spermatogenesis can be divided into three major phases: mitotic process of stem cells (spermatogonia) to form spermatocytes, meiosis to reduce the number of chromosomes to form spermatids, and spermiogenesis in which haploid spermatids develop into spermatozoa [1], [2]. Among these, meiosis and haploid stages are unique to germ cells, and often require specific genes to execute unique regulatory roles [3]. The patterns of gene expression in meiotic and haploid germ cells could be repressed by post-transcriptional control [3]. This repression is partly achieved by some regulatory elements through binding mRNA un-translated regions [3], [4]. Some regulatory small RNAs including small interfering RNAs (siRNAs), PIWI-interacting RNAs (piRNAs) and microRNAs (miRNAs) have emerged as important regulators of eukaryotic gene expression and have been used in elucidating the molecular mechanisms regulating spermatogenesis [5], [6].

piRNAs have been recently discovered from mammalian testes [7]–[9]. piRNAs are longer (26–32 nt) than miRNAs and siRNAs (21–23 nt) and bind to PIWI, a spermatogenesis-specific protein belonging to the Argonaute protein family [7]–[9]. Mammalian piRNAs can be divided into pre-pachytene (26–28 nt) and pachytene (29–31 nt) piRNAs [10]. Pachytene piRNAs appear around the pachytene stage of meiosis, become exceptionally abundant, and persist until the haploid round spermatid stage, after which they gradually disappear during sperm differentiation; while pre-pachytene piRNAs are expressed in spermatogonia before meiosis and become depleted starting at mid-pachytene [11]. piRNA biogenesis is not yet fully understood, although there are two popularly accepted mechanisms. In somatic cells, piRNAs are produced through a PIWI-dependent, AUB- and AGO3-independent pathway, whereas in the germline, piRNAs are generated through an AUB- and AGO3-dependent piRNA amplification cycle [12]. This amplification cycle is also called as the “Ping Pong” mechanism wherein an antisense primary piRNA binding with PIWI and AUB recognizes a sense transposon transcript, and causes the recruitment of AGO3, and then results in the cleavage of the transcript at a point ten nucleotides from the 5′ end of the primary piRNA, producing the sense secondary piRNA; the mature sense piRNA then guides cleavage of the antisense transposon transcript, thus additional copies of the original antisense piRNA are generated [13], [14]. Besides PIWI, other proteins such as Rhino (RHI) [15], Maelstrom (MAEL) [16], Germ cell protein with Ankyrin repeats, Sterile alpha motif, and leucine Zipper (GASZ) [17] are also required for biogenesis and/or stability of piRNAs.

piRNAs have a specific germline function in repressing transposons and other repetitive elements by involving heterochromatin formation and transcriptional and post-transcriptional silencing [13], [18], [19]. The piRNA pathway protects the germline genome from DNA damage and mutation, ensuring that genetic information passes faithfully from generation to generation [14], [20]. In addition, the great number of piRNAs specifically appeared in the testis, suggesting their essential functions in meiosis and spermatogenesis [2]. However, the precise function of piRNAs in germ cells still remains unknown. Identification of abundant piRNAs in male germ cells is a prerequisite for a thorough understanding of their roles in regulating spermatogenesis. We therefore constructed and sequenced a small RNA library prepared from three sexually mature Large White testes. The library generated 6,913,561 clean short reads, from which piRNA candidates (≥26 nt) were predicted by a *k*-mer scheme and by homology searches, respectively. We selected 7 piRNAs identified by homology searches to perform their expression pattern. Our present research significantly enhances our knowledge of the porcine piRNAs and indicates their potential functions.

## Results

### An Overview of the Sequenced Small RNAs from the Pig Testes

**Figure 1:**
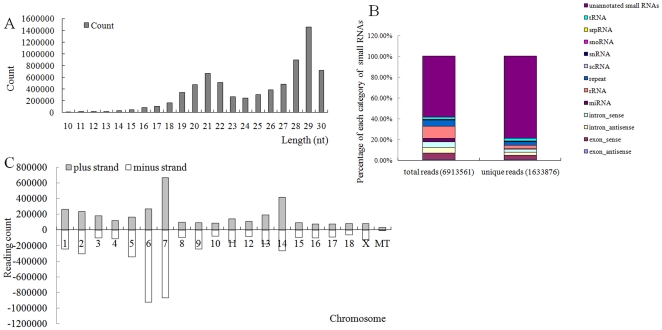
Characterization of *sus scrofa* small RNAs. (A) Length distribution of small RNAs. *Sus scrofa* small RNAs displayed a bimodal length distribution with two peaks at 21 nt and 29 nt. (B) Bar chart summarizing the annotation of small RNA populations in total RNA from testes. (C) Chromosomal distribution of small RNAs.

In order to identify the porcine piRNAs, a small RNA library of 3 sexually mature porcine testes was sequenced using Solexa technology. We obtained 9,533,729 of raw sequence reads which comprised 1,961,666 low-quality reads (20.58%) and 7,572,063 high-quality reads (79.42%). Of high-quality reads in this library, 6,913,561 (91.30%) clean reads of *Sus Scrofa* small RNA fraction (18–30 nt) were used to map the swine genome assembly using the Short Oligonucleotide Analysis Package (SOAP), leading to 4,527,258 genome-matched reads (Table S1). Pig small RNAs demonstrated a bimodal length distribution with two peaks at 21 nt and 29 nt (Figure 1A). Sequence analysis of cloned small RNAs with a length range of 18–30 nt indicated that 68.44% (1,118,158/1,633,876) contained a 5′ uridine residue. Subsequently, all clean reads of at least 18 nt were divided into different categories of small RNAs according to their biogenesis and annotation (Figure 1B). The significant fraction (14.16%) of the total clean reads of at least 18 nt was derived from putative degradation products of rRNAs, tRNAs, small nuclear RNAs and other non-coding RNAs. Substantial portions (10.97% and 6.74%) matched the intronic and exonic regions of protein-coding genes, respectively. About 6.01% and 3.44% were finally screened as highly repeated sequences, and miRNA candidates, respectively. The largest fraction (58.68%) was from un-annotated genomic sites. The small RNAs isolated here were located majorly on *Sus scrofa* chromosome (SSC) 1, 2, 5–7 and 14 (Figure 1C).

### Prediction of piRNAs from the Repertoire of Small RNAs by a *k*-mer Scheme

938,328 small RNA unique reads of 26–30 nt were obtained and the candidate piRNAs were predicted by a *k*-mer scheme using online piRNApredicator (http://59.79.168.90/piRNA/analysis.php). We got 375,195 piRNAs diversely distributed among rRNAs, tRNAs, snRNAs, snoRNAs, repeat, genes and un-annotated regions (Table S1). Accordingly, the 34,482 unique piRNAs (9.20%) were generated from genes, and only 3.38% of the unique piRNAs were mapped to repeat sequences. However, the largest fraction (84.67%) of piRNAs was not annotated. The predicted piRNAs had signature piRNA characteristics, including a preference for a uridine (U) at their 5′ end (79.05%). But we noted that only a small amount of piRNAs (29.62%) carried a conserved A at position 10.

### Repeat-Derived piRNAs

In the present study, repeat-derived piRNAs only constituted 3.38% of unique reads and 9.47% of total reads of all predicted piRNAs (Table S1). About 73.06% (9263) and 15.89% (2015) of repeat piRNAs derived from long interspersed nuclear elements (LINEs) and long terminal repeats (LTRs), respectively. There was not a strand preference in the distribution of repeat-derived piRNAs (6044 sense vs 6409 antisense) (Table S2). LINE piRNAs were equally divided between sense (4652 unique piRNAs) and antisense (4611 unique piRNAs) sequences, while LTR piRNAs were preferentially distributed on antisense strands (848 sense vs 1167 antisense) (Table S2).

### Gene-Derived piRNAs

Gene-derived piRNAs constituted 9.2% of all piRNAs categories (Table S1). The minority (31.63%) of gene-derived piRNAs was mapped to exons of mRNAs, and 68.37% were mapped to introns, strongly suggesting the gene-derived piRNAs are generated from primary transcripts. To fully investigate the function of gene-derived piRNAs, we collected transcript IDs and performed a Gene Ontology (GO) term and Kyoto Encyclopedia of Genes and Genomes (KEGG) pathway annotation using the DAVID gene annotation tool (http://david.abcc.ncifcrf.gov/). Totally 34,482 gene-originated piRNAs were generated from 5547 (1688 *Sus scrofa*, 4089 unknown) unique genes. The enrichment of functional annotation terms (FATs) of these unique genes was analyzed using DAVID (http://david.abcc.ncifcrf.gov/). FATs with enrichment score >1 were regarded as enriched (Table S3). Class 1 of piRNA-generating genes were enriched with FATs such as nucleosome assembly, nucleosome organization, cellular macromolecular complex assembly, chromatin assembly or disassembly, chromatin organization, DNA binding, suggesting that the proteins of this cluster were mainly involved in regulation of gene expression. Class 1 included some histone genes, such as histone cluster 1, H1d (*HIST1H1D*), histone cluster 1, H1c (*HIST1H1C*), histone cluster 1, H4b (*HIST1H4B*), histone cluster 1, H4j (*HIST1H4J*) coding proteins which participated in chromatin remodeling, DNA repair and genome maintenance [21]. KEGG gene pathway analysis showed that piRNA-generating genes played important roles in 13 pathways including Systemic lupus erythematosus, Alzheimer’s disease, Huntington’s disease, Parkinson’s disease, Oxidative phosphorylation. Interestedly, most of gene pathways were associated with disease (Table S4).

### Chromosomal Distribution and piRNA Gene Clusters

All of the 375,195 piRNAs predicted by the *k*-mer scheme were aligned against the pig genome (Sscrofa9 (April 2009) assembly) using SOAP program. Only sequences that perfectly matched the pig genome along their entire length were considered for further analysis. Consequently, 238,700 (63.62%) were perfectly mapped to 3,723,339 locations in the draft assembly of the *Sus scrofa* genome. Of the perfectly matching piRNAs, 197,673 were mapped to only a single location, and 41,027 were mapped to multiple genomic locations (average 85.93 locations).

piRNA-coding sequences displayed a highly uneven distribution among chromosomes. piRNAs were enriched on SSC5, 11, 14 with more than 100 piRNAs per 1 Mb genomic region, but were sparse on SSC 8–10, 15–18, X with less than 40 piRNAs per 1 Mb genomic region (Table 1). Taken SSC7 and SSCX as examples, SSC7, representing only 6.03% of the genome, encoded 23.03% of the piRNAs (Table 1), while SSCX, representing 5.5% of the genome, contained 0.5% of piRNA sequences, 10- fold lower than the expected value for random distribution. Mitochondria DNA (mtDNA) was the densest piRNA-populated region with 11978.57 piRNAs per 1 Mb genomic sequence.

**Table 1:** The chromosomal distribution of piRNAs and piRNA clusters in the genome.

Chromosome	piRNA*		Cluster		Chromosome		Density	
	Count	Percentage (%)	Count	Percentage (%)	Size (Mb)	Percentage (%)	piRNA/Mb	Cluster/10 Mb
1	17866	9.04	102	9.07	295.54	13.06	60.45	3.45
2	12109	6.13	90	8.01	140.14	6.19	86.41	6.35
3	7438	3.76	94	8.36	123.61	5.46	60.18	7.60
4	4482	2.27	65	5.78	136.26	6.02	32.89	4.77
5	17283	8.74	44	3.91	100.52	4.44	171.93	4.38
6	20451	10.35	114	10.14	123.31	5.45	165.85	9.24
7	57391	29.03	119	10.59	136.41	6.03	420.71	8.58
8	2038	1.03	26	2.31	119.99	5.30	16.98	2.17
9	4017	2.03	57	5.07	132.47	5.85	30.32	4.30
10	2680	1.36	32	2.85	66.74	2.95	40.15	4.79
11	8210	4.15	35	3.11	79.82	3.53	102.86	4.38
12	3565	1.80	64	5.69	57.44	2.54	62.07	11.14
13	8918	4.51	52	4.63	145.24	6.42	61.40	3.58
14	21853	11.06	98	8.72	148.52	6.56	147.14	6.60
15	2973	1.50	34	3.02	134.55	5.95	22.10	2.53
16	1102	0.56	15	1.33	77.44	3.42	14.23	1.94
17	2603	1.32	48	4.27	64.40	2.85	40.42	7.45
18	1478	0.75	25	2.22	54.32	2.40	27.21	4.60
X	1017	0.51	9	0.80	125.88	5.56	8.08	0.71
Mt	199	0.10	1	0.09	0.017	0.0007	11978.57	601.94
Total	197673	100	1124	100	2262.596	100	87.37	4.95

* All piRNAs with the unique perfectly mapping to pig genome were used to analyze to chromosome distribution. MT, mitochondrial genome.

**Figure 2:**
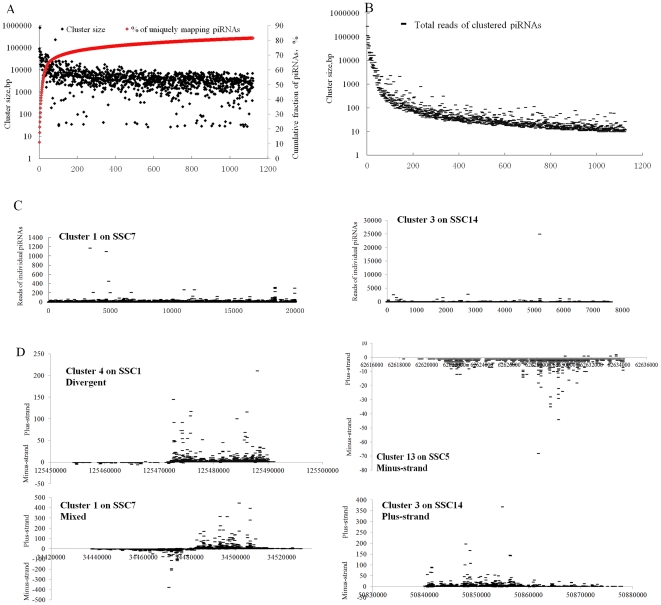
Characterization of *sus scrofa* piRNA clusters. (A) 1124 piRNA clusters were identified by scanning 10 kb genomic loci that produced at least 10 uniquely mapping piRNAs, and were thus ranked by the number of uniquely mapping piRNAs that they produced. Black diamonds and red curve show the genomic size of each cluster and the cumulative fraction of piRNAs contributed by clusters, respectively. (B) piRNA cluster expression were calculated as total reads of piRNAs within a cluster. (C) Cluster 1 on SSC7, and Cluster 3 on SSC14 were taken as the examples to describe the expression levels of individual piRNA in a cluster. (D) There were four types of piRNA clusters including divergent, plus-strand, minus-strand, and mixed type according to the definition of piRNA cluster type described by Lau et al. (2006).

To investigate the genomic origin of pig piRNAs, we searched for uniquely mapping sequences in close proximity in the genome. Using a threshold value of 10 piRNAs per 10 kb as previously described [11], we identified 1124 clusters which were ranked by their relative contributions to piRNA populations (Table S5). The most prominent cluster on SSC7 contained 11.13% of all uniquely mapping piRNAs, and the individual contribution of each subsequent cluster dropped dramatically (Figure 2A, Table S5). Each cluster contained 10–22002 piRNAs (average 143.93 piRNAs) and spanned 27–90603 bp (average 5206.9 bp), and combining all clusters yielded about 5.85 Mb of genomic space, which could accommodate 81.61% of all 197,673 uniquely mapping piRNAs (Figure 2A, Table S5). The cluster expression, measured by the total reads of uniquely mapping piRNAs within a cluster, shared a very similar pattern with the number of uniquely mapping piRNAs (Figure 2A, 2B). And generally piRNAs within a same cluster had a relatively similar expression (Figure 2C).

According to the definition of piRNA cluster type described by Lau et al. (2006) [9], here 47.69% (536) and 44.57% (501) piRNA gene clusters were on the minus and the plus strands, respectively (Table S5). And 48 clusters distributed on two strands but in a divergent, nonoverlapping manner (Table S5). Only 39 mixed type clusters had hits that suggested convergent or overlapping transcription (Table S5). The most prominent cluster (Cluster 1 on SSC7) showed a mixed type which contained regions of minus- and plus-strand hits that were overlapped with each other (Figure 2D). Cluster 3 on SSC14 and Cluster 13 on SSC5 showed a profound strand asymmetry, with the vast majority of piRNAs being derived from one genomic strand (Figure 2D). Cluster 4 on SSC1 was a bidirectional cluster with a divergent, bidirectional transcription orientation (Figure 2D).

### Homology Searches for Porcine piRNAs and their Expression Patterns

We used BLASTN to identify piRNA candidates by aligning 938,328 Solexa deep sequences of more than 26 nt with piRNA sequences in the RNAdb2.0, even though piRNAs were poorly conserved between distant species. Only 326 small RNAs have (a) perfectly matched homological piRNAs (Table S6), confirming that individual piRNA sequence was poorly conserved. However, there were still some piRNAs with a high similarity with other species. For example, t0000740 had 100% similarity with mature sequences of hsa_piR_004153, ona_piR_166322 and rno_piR_001199 (Table S7).

**Figure 3:**
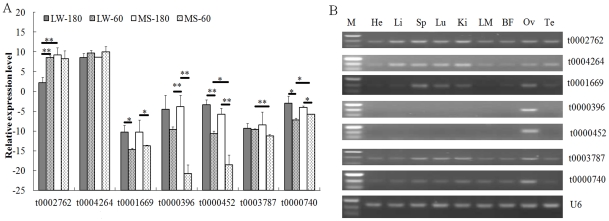
Seven piRNAs expression profile in the porcine testes. (A) Seven piRNAs expression profile in the porcine testes from 60-day and 180-day testes of Chinese Meishan and Large White pigs using stem-loop qRT-PCR method. The X-axis represents the small RNAs and the Y-axis shows the relative expression levels of small RNAs (–ΔC_t_ values for qRT-PCR). The significance of differences for the expression between 60-day (sexually immature) and 180-day (sexually mature) testes of Chinese Meishan and Large White pigs was calculated using two-tailed T-test. *, p≤0.05; **, p≤0.01. (B) Seven piRNAs expressed in the porcine testes by stem-loop semi-quantitative RT-PCR. M, 25 bp DNA ladder; He, heart; Li, liver; Sp, spleen; Lu, lung; Ki, kidney; LM, *longissimu*s *dorsi* muscle; BF, backfat; Te, testis; Ov, ovary.

Seven pig piRNAs including t0002762, t0004146, t0001669, t0000396, t0000452, t0003787, t0000740 were detected by homology searches through aligning to piRNA sequences in piRNA banks [22]. Using a stem-loop quantitative RT-PCR analysis, we investigated the relative expression levels of these 7 piRNA candidates in the testes of Large White and Chinese Meishan at the age of 60 days and 180 days (Figure 3A). Five piRNAs (t0001669, t0000396, t0000452, t0003787, t0000740) were up regulated in testes of 180 days compared with 60 days. And then a semi-quantitative RT-PCR was used to validate their germ-specific expression pattern of these 7 piRNAs. The relative abundance of piRNAs in all 9 analyzed tissues followed similar patterns, showing the highest expression levels in testes and lowest levels in heart, *longissimus dorsi* muscle and backfat (Figure 3B). Five small RNAs (t0002762, t0004146, t0001669, t0003787, t0000740) displayed a ubiquitous expression pattern, while t0000396 and t0000452 were testis-specific (Figure 3B).

## Discussion

piRNAs are a new class of small RNAs that bind a spermatogenesis-specific protein belonging to the Argonaute protein family called PIWI [7], [9]. The initial identification of piRNAs was made by cloning from the PIWI immunoprecipitants [9], [23], [24], or by cloning from the RNA testicular library of *PIWI* knockout mice [7]. Some small RNAs called as “piRNA-like RNAs (pilRNAs)” were cloned directly from the testicular RNA library without testing their interaction with PIWI, and then their expression in multiple tissues were detected to validate their authenticity according to the testes-specific nature of piRNAs [2]. Using this small RNA cloning method, 630 pilRNAs were cloned from the mouse testis, among which only 89 pilRNAs matched known piRNAs, suggesting that thousands of piRNAs remain to be identified in the testis [2].

Solexa is a breakthrough sequencing technology which enables high throughput short sequencing reads to be generated in one run, and avoids the bacterial cloning steps in Sanger sequencing [25]. Here Solexa deep sequencing was used to get 6,913,561 clean reads, which was much higher throughput than the conventional cDNA cloning method. And then two different *in silico* predictions (a *k*-mer scheme and homology searches) were used to identify the porcine piRNAs. Due to lack of conservation of piRNA sequences among different species, the homology search methods including BLAST are inappropriate for piRNA prediction. For example, Xie et al (2011) only identified 89 piRNAs from Solexa sequences of a pool of equal amounts of RNA from 16 different porcine tissues [26]. In the present study, we used BLAST to align 938,328 Solexa deep sequences of more than 26 nt with piRNA sequences in the RNAdb2.0, and then only found 326 small RNAs had (a) perfectly matched homological piRNAs. Fortunately, a *k*-mer scheme was developed using a new all the 1364 1–5 nt strings and an improved Fisher Discriminant algorithm to characterize piRNA sequences in rats, mice, human beings, fruit flies and nematods [27]. Using this algorithm, we identified 375,195 candidate piRNAs from 938,328 small RNA unique reads of 26–30 nt.

piRNAs are diversely distributed among exonic, intronic, intergenic, and repeat sequences [9], [23], [24]. Here the predicted piRNAs were enriched un-annotated regions that were poor in genes or repeats. Only 34,482 (9.20%) piRNAs were generated from genes, similar to the report in mice with 9.3% within introns of known genes and 1.3% in exons [7]. Only 3.38% of piRNAs were mapped to repeat sequences, noticeably lower than the presence of such sequences in 42.3% of the whole genome, and also lower than the presence of piRNAs in repeat sequences in rat, *Drosophila* and mice (15–21%) [9], [23], [24]. The predicted piRNAs had a strong preference for a uridine (U) at their 5′ end (79.05%). However, an amount of piRNAs (70.38%) did not carry a conserved A at position 10, consistent with the observation that this class of piRNAs was pachytene piRNA as they outcompeted pre-pachytene piRNAs in small RNA cloning from the sexually mature testes [7], [11], [23], [24].

The GO analysis of piRNA-generating genes showed that Class 1 was enriched with FATs such as nucleosome assembly, nucleosome organization, cellular macromolecular complex assembly, suggesting that the proteins of this cluster were mainly involved in regulation of gene expression. Interestedly, some histone genes including *HIST1H1D, HIST1H1C*, *HIST1H4B*, *HIST1H4J* were assigned to Class 1. During spermatogenesis in mammals, an extensive change in chromatin structure and H1 histone gene expression occurs [28]. In spermatogonia the histone subtypes such as Hlb-Hle can be detected during all stages of spermatogenesis up to the primary spermatocyte stage [29]. However, in mice the pachytene piRNAs have an abundant expression from pachytene spermatid stage to haploid round spermatid satge [11]. The negative relationship of the expression patterns between piRNAs and their generating mRNAs in the spermatogenesis stages [30] suggests the histone genes are the potential origins of the pachytene piRNAs.

KEGG gene pathway analysis showed that piRNA-generating genes played important roles in several associated-disease pathways including Systemic lupus erythematosus, Alzheimer’s disease, Huntington’s disease (Table S4). Recently, piR-823 demonstrated in vitro and in vivo tumor suppressive activity in human gastric cancer cells [31]. piR_015520, located in intron 1 of the human Melatonin receptor 1A (*MTNR1A*) gene, was detected in the brain where deregulation of the *MTNR1A* occurred in neurodegenerative diseases such as Alzheimer’s disease [32]. Therefore, piRNAs are potentially involved in diseases by regulating disease-correlated mRNAs.

piRNAs show a rather uneven distribution among chromosomes [7], [23], [24]. In our study, they were enriched on SSC5, 11, 14 but were scattered sparsely on SSC 8–10, 15–18, X. SSCX contained 0.5% of piRNA sequences, similar with the density of piRNAs (0.4%) in mice [7], 10 fold lower than the expected value for random distribution. mtDNA had 11978.57 piRNAs per 1 Mb genomic sequence, more densely populated by piRNAs than nuclear genomic DNA. Although researchers have not identified the role of piRNAs in mitochondria, the activity of a mitochondrial enzyme-zuc/MitoPLD is required for piRNA mediated silencing of transposable elements in fly and mouse germlines, suggesting that signaling from mitochondria influences the piRNA pathway [33]–[35].

piRNAs are derived from clustered loci which play an important role in piRNA generation [9], [11], [13], [23]. 1124 clusters were identified using a threshold value of 10 piRNAs per 10 kb as previously described [11], were then ranked by their relative contributions to piRNA populations (Table S5). The most prominent cluster, containing 11.13% of all uniquely mapping piRNAs, was on SSC7 (Figure 2A, Table S5). And generally piRNAs within a same cluster had a relatively similar expression (Figure 2C), as the highly clustered organization raises the possibility that the piRNAs within each cluster are coordinately expressed and might share related functions [7]. According to the definition of piRNA cluster type described by Lau et al. (2006) [9], here we designated 536, 501, 48 and 39 piRNA clusters as minus type, plus type, divergent type and mixed type, respectively (Table S5). The divergent type contained regions of minus- and plus-strand hits that were juxtaposed with each other but separated by a gap, an orientation that suggested divergent, bidirectional transcription, and these bidirectional clusters had been postulated to share the same central transcriptional promoters [24].

Spermatogenesis involves many cellular and molecular events unique to germ cells, such as meiosis and spermiogenesis. In Meishan boars, leptotene stage spermatocytes, round spermatids and spermatozoa were first found in the section of seminiferous tubules at 30–45, 60 and 75 days of age, respectively; after 105 to 120 days of age, most testicular spermatozoa were morphologically normal [36]. The formation of the first spermatozoa occurred 2 months earlier in Meishan than in European breeds [37]. Non-coding small RNAs execute the regulatory roles in the spermatogenesis which require germ cell specific mRNAs. Here five piRNAs (t0001669, t0000396, t0000452, t0003787, t0000740) were up regulated in testes of 180 days compared with 60 days, suggesting that piRNAs are mainly expressed in spermatids. Our result of expression pattern of piRNAs was consistent with the result of Ro et al. (2007) which found that the largest number and the highest levels of pilRNA were detected in purified spermatocytes and round spermatids [2].

In the initial reports, piRNAs have been shown to be expressed exclusively in the testis [7], [23], [24]. However, we found that 5 of 7 tested piRNAs had a uniquious expression (Figure 3B). Consistent with our results, Ro et al. (2007) found only 30% of pilRNAs cloned were predominantly expressed in the testis [2]. Afterwards, piRNAs have been found outside of the testis, such as in the ovary [38], [39], in the central nervous system [40] and in human gastric cancer cells [31], suggesting piRNAs have a potential function on other biological processes besides on germ development. Testis-specific t0000396 and t0000452 belonged to repeat LINE/L2∶0 and un-annotated small RNAs, respectively. Therefore, these 2 piRNAs could be the piRNA candidates due to their testis-specific nature (Figure 3B) and their up-regulation in sexually mature testes (Figure 3A). Their function on spermatogenesis will be investigated in our future work.

## Materials and Methods

### Ethics Statement

This study was approved by the ethics committee of Huazhong Agricultural University (No.30700571) [3].

### Animals

Animals used for the Solexa deep sequencing were three Large White boars of 180 days. Totally 12 animals used for small RNAs expression profile included 3 boars of 60-day Chinese Meishan, 180-day Chinese Meishan, 60-day Large White, and 180-day Large White, respectively. All animals were from the pig farm of Huazhong Agricultural University (Wuhan, China). Some tissues were removed from animals and immediately snap-frozen in liquid nitrogen and stored at –80°C. RNAs were isolated from 9 different porcine tissues (heart, liver, spleen, lung, kidney, *longissimus dorsi* muscle, backfat, testis, ovary) from postnatal day 120 of the Large White pigs.

### Construction and Sequencing of a Small RNA Library

We employed Illumina sequencing methods as described previously [29]. Briefly, total RNAs of three 180-day porcine testes were extracted by Trizol reagent (Invitrogen) according to manufacturer’s protocols. The small RNAs were size-fractionated from the RNA pool of three samples, purified by polyacrylamide gel electrophoresis to enrich for molecules in the range 18–30 nt, and ligated to 5′- and 3′- end RNA oligonucleotide adaptors. cDNA constructs were created by RT-PCR based on the small RNAs ligated with 3′ and 5′ adaptors. The purified PCR products were loaded on the Illumina Cluster Station. All sequencing was carried out at the Beijing Genomics Institute (BGI), Shenzhen. Raw data from Illumina 1G Genome Analyzer were processed using the Solexa software (Illumina). Low quality reads were filtered according to the base quality value. 3′ adaptor sequences were accurately trimmed using a dynamic programming algorithm and 5′ adaptor contaminants were removed. Adaptor sequences were accurately clipped using a dynamic programming algorithm.

### Classification and Functional Annotation of Sequenced Small RNAs

After redundancy was removed, sequences ≥18 nt were analyzed to get their length distribution by an in-house software developed by BGI (Shenzhen). Then the small RNAs were perfectly mapped to the swine genome using SOAP. Pig small RNAs were functionally annotated using the online databases including swine genome database (Sscrofa9.0, Apr 2009), human repeat database (hg18 database), miRBase release 14.0, GenBank, Rfam (9.1). Sequences that gave hits for known non-coding RNAs (ncRNAs) were classified as “ncRNA” and those that gave hits for genic regions (exons) were classified as “gene”. We classified the remaining small RNAs as “no annotation”. Small RNAs ≥ 26 nt were used to predict piRNA sequences by online piRNApredictor software (http://59.79.168.90/piRNA/analysis.php), a novel ‘dynamic’ algorithm with a precision of over 90% and a sensitivity of over 60% [27].

### Enrichment Analysis of GO Functions and Gene Pathways

The DAVID functional annotation tool (http://david.abcc.ncifcrf.gov/) was used to perform GO classification and pathway annotation of piRNA-generating mRNAs [41]. Functional annotation terms from the ontologies of “biological processes” and “molecular function” were recorded with EASE threshold 0.1 and count threshold 2 [42]. The enrichment score cutoff was set to 1.0. The genes which generate piRNAs were assigned to pathways analysis using the online DAVID.

### Homology Searches for piRNAs and their Expression Profile by RT-PCR

In order to identify piRNAs in pigs, we used BLASTN to analyze alignments of 938,328 Solexa deep sequences of more than 26 nt with over 88,000 mouse, human and rat piRNA sequences in the RNAdb2.0 (http://research.imb.uq.edu.au/rnadb/) [43]. Only candidate piRNAs with perfectly mapping matches were listed in Table S6. Expression patterns of seven piRNAs identified by aligning to piRNA sequences in piRNA banks were investigated using a stem-loop real-time RT-PCR as described by Chen et al. (2005) [44]. Total RNA was isolated from 3 independent 60-day testes, 180-day testes from Chinese Meishan and Large White boars. cDNA was synthesized in a 20 µl reverse transcription (RT) reaction with 1 µg purified total RNA, 10 µl 2 × TS Reaction Mix (Transgen), 1 µl TrasScript RT/RI Enzyme Mix (Transgen), 0.01 µM piRNA-specific stem-loop primers and 0.01 µM reverse primer U6-R (Table S7). RT reaction mixture was incubated at 42°C for 30 min, inactivated at 85°C for 5 min.

Real-time PCR was performed using a standard SYBR Green PCR kit (Toyobo) on the Roche LightCycler480 Real-time PCR Detection System. A 25 µl real-time PCR reaction contained 12.5 µl 2 × SYBR Green Real-time PCR Master Mix (Toyobo), 0.3 µM piRNA-specific forward primer and universal reverse primer (Table S7) and 0.5 cDNA products. And the reaction mixture was incubated at 95°C for 2 min, then 40 cycles for 20 sec at 95°C, 15 sec at 60°C and 15 sec at 72°C, 1 cycle for 30 sec at 95°C and 30 sec at 58°C, continuous at 95°C, finally hold at 42°C. Porcine *U6* was used as an internal control and all reactions were run in triplicate. Due to the negative relationship between Ct and expression level, Student’s t-test was conducted to identify differentially expressed piRNAs by comparing the –ΔCt values of qRT-PCR of two groups [4]. The significant level was set to 0.05.

Stem-loop semi-quantitative RT-PCR was used to detect the piRNA expression pattern in 9 porcine tissues including heart, liver, spleen, lung, kidney, *longissimus dorsi* muscle, backfat, testis, ovary, as described previously [45]. The reaction mixture was incubated at 95°C for 4 min, then 30 cycles for 30 sec at 94°C, 30 sec at 60°C, 15 sec at 72°C, 1 cycle for 10 min at 72°C. Semi-quantitative PCR analyses were performed such that the PCR cycle numbers were empirically determined to ensure that each of the amplification reactions was in the exponential range.

### Conclusion

In conclusion, we reported here the discovery of 375,195 piRNA candidates by a *k*-mer scheme and 326 piRNA candidates by homology searches from 938,328 small RNAs sequenced by next generation method (26–30 nt). The ubiquitous expression profiles for the novel piRNAs indicated that the roles of piRNAs were not limited to spermatogenesis as the previous studies suggested. Furthermore, transfection experiments will be done to test the ability of piRNAs to bind their genomic regions, thereby affecting their host gene expression. The newly identified piRNAs from the pig testes significantly enhance our knowledge of small RNAs species in pigs.

## Supporting Information

Table S1 **The flowing results of data filtration and the distribution of sequenced small RNAs from pig testes.** After sequential filtration, the raw data of pig testes library were separated into low and high quality reads. High quality reads with at least 18 nt were differentiated into categories of short sequencing reads, including rRNA, tRNA, snoRNA, fragments of sense and antisense exons and introns of coding genes, repeat sequences in transposable elements, unannotated short sequencing reads and some annotated miRNAs. Novel miRNAs were screened from unannotated sequencing reads.). Small RNAs ≥ 26 nt were used to predict piRNA sequences by online piRNApredictor software (http://59.79.168.90/piRNA/analysis.php).
(XLS)

Table S2 **List of transposable elements and the related repeat sequences which generate small RNAs.**
(XLS)

Table S3 **Go terms of piRNA-generating genes with the threshold of enrichment score 1.**
(XLS)

Table S4 KEGG pathways annotation of piRNA-generating genes predicted by online DAVID software.
(XLS)

Table S5 **The cluster distribution of piRNAs in the genome.** piRNA clusters were defined into four types (divergent, plus-strand, minus-strand, and mixed) by the following algorithm. Each clusters was scanned first on the plus strand (from the left boundary to the right boundary) and sequentially on the minus strand (from the right boundary to the left boundary) for 5 consecutive loci where reads were mapped uniquely. Searches that identified 5 consecutive loci only from one strand in a cluster logically classified the cluster as either a plus-strand or minus-strand type. If 5 consecutive loci were identified on both the plus- and minus-strand searches, and the plus-strand loci were located downstream of minus-strand loci, such a cluster would then be classified as a divergent type. In other cases, the cluster is classified as mixed type (Lau et al., 2006).
(XLS)

Table S6 **Homology search of pig piRNAs against piRNA database.** BLASTn was used to analyze alignments of the Solexa deep sequences of more than 26 nt with human, mouse and rat piRNA sequences downloaded from RNAdb.
(XLS)

Table S7 **Primer pairs used to investigate the expression profiles of pilRNAs by quantitative RT-PCR.**
(XLS)
